# Socioepidemiologic Profile and Treatment-seeking Behaviour of HIV/AIDS Patients in a Tertiary-care Hospital in South India

**Published:** 2014-12

**Authors:** Abyramy Balasundaram, Sonali Sarkar, Abdoul Hamide, Subitha Lakshminarayanan

**Affiliations:** ^1^Department of Preventive and Social Medicine, JIPMER, Puducherry 605 006, India; ^2^Department of Medicine, JIPMER, Puducherry 605 006, India

**Keywords:** Acquired immunodeficiency syndrome, Epidemiology, Health behaviour, Time to treatment, India

## Abstract

India has the third largest number of people living with HIV/AIDS. Provision of free antiretroviral therapy (ART) for eligible persons living with HIV (PLHA) has been scaled up significantly both in terms of facilities for treatment and number of beneficiaries. This study aimed at describing the profile of HIV/AIDS patients on ART from a tertiary-care hospital and to explore the factors associated with treatment-seeking behaviour, family support, and perceptions regarding HIV and ART. This is a descriptive study conducted at the ART centre in a tertiary-care hospital in Puducherry. Study population consisted of 130 HIV-positive patients aged more than 18 years on free firstline ART for at least 6 months. Data on sociodemographic details, clinical details, treatment-seeking behaviour, family support, and perceptions regarding HIV and ART were collected using a pretested questionnaire. Data are presented as percentages. In total, 130 patients on ART for at least 6 months were included in the study—61% were males (n=79), 39% were females (n=51); half of them belonged to the age-group of 36-50 years. Half of the participants were diagnosed to have HIV/AIDS between 1 and 3 year(s); two-thirds had one or more co-infection(s). The majority were aware of the side-effects of ART. After advice to start ART, there was a delay in starting treatment in one-fifth of the subjects due to depression, fear of stigma, disclosure to family, and side-effects. More than two-thirds of the patients travelled more than 30 km distance. Families of HIV-positive subjects were supportive in accompanying to the ART centre, collecting drugs, reminders to take medication, and motivation to complete the treatment. Alcohol (50%) and tobacco consumption (39%) was common among the subjects. Half of the respondents stated stigma, death, and pain as the main fears, and all of them stated high levels of trust and rapport with their doctors. This study reveals several positive aspects among ART beneficiaries. However, issues, like tobacco and alcohol consumption, travelling long distance for drug collection, fear of stigma and death, and concerns regarding the future, need to be addressed.

## INTRODUCTION

India has the third largest number of people living with HIV/AIDS in the world (1). According to the National AIDS Control Organization (NACO), the HIV prevalence among adults at the national level has continued its steady decline from 0.41% in 2000 to 0.31% in 2009. It is estimated that 2.39 million people are infected with HIV in India, of whom 39% are female, and 4.4% are children (1).

Provision of free antiretroviral therapy (ART) for eligible persons living with HIV (PLHA) was launched on 1 April 2004 in eight government hospitals located in six high-prevalence states. Since then, the programme has been scaled up significantly both in terms of facilities for treatment and the number of beneficiaries seeking ART. ART centres are established at the department of medicine in medical colleges and district hospitals in the government sector. There are 355 ART centres and 725 link ART centres in India, with around 1.5 million PLHA registered as of March 2012 (2). HIV prevalence among adults in Tamil Nadu was 0.33%, with 43 ART centres and 85 link ART centres. Tamil Nadu had 154,742 registered PLHA, with 61,473 of PLHA on the firstline ART (cumulative).

Puducherry, a Union Territory situated 165 km from Chennai, the capital of Tamil Nadu, has a population of 1.24 million and an HIV prevalence of 0.28% (3). Pondicherry district belonging to the Union Territory of Puducherry is surrounded by Villupuram and Cuddalore districts of Tamil Nadu state (4). The HIV clinic at Jawaharlal Institute of Postgraduate Medical Education and Research (JIPMER), a teaching tertiary-care referral hospital, caters to patients from Puducherry and a wide area bordering Vellore, Cuddalore, Villupuram, and Tiruvannamalai districts of Tamil Nadu.

To facilitate the delivery of ART services nearer to the beneficiaries, it was decided to set up link ART centres located mainly at Integrated Counselling and Testing Centre (ICTC) in the district/subdistrict-level hospitals nearer to the patient's residence and linked to a nodal ART centre within accessible distance. Presently, 725 link ART centres have been established and made functional as of March 2012 (2). The scaling-up of access to ART has benefitted PLHA tremendously. Data on HIV healthcare delivery from different settings and geographic areas are essential to optimize HIV care. This study aims at understanding the characteristics of beneficiaries of ART clinic of a tertiary-care hospital and to explore the factors associated with treatment-seeking behaviour, family support, and perceptions regarding HIV and ART.

## MATERIALS AND METHODS

### Study setting

This is a descriptive study conducted over a period of five months (June to October 2012) at the link ART centre in JIPMER, a tertiary-care hospital in Puducherry.

### Participants

Study population consisted of 130 HIV-positive patients aged more than 18 years on the free firstline ART from JIPMER for at least 6 months.

### Ethical considerations

The study was approved by the Scientific and Ethics Committee of the JIPMER. Confidentiality of collected data was ensured to the participants. The subjects were approached by the investigator, and informed consent was obtained for inclusion in the study. Those who gave consent for the study were interviewed with a pretested structured questionnaire.

### Data-collection tool

The questionnaire included the following sociodemographic details: age, gender, education, occupation, socioeconomic status, family composition, and place of residence. Modified Kuppuswamy's classification (2011) was used in order to classify the study population into 5 classes of socioeconomic status (Table 1). Collected clinical details included treatment received, side-effects, and health status. Treatment-seeking behaviour of these subjects was explored as reasons for delay in the initiation of ART, reasons for preferring the centre, accompanying persons, distance of the place of residence from the centre, mode of travel, and money spent. Family support to these subjects and perceptions regarding HIV and ART were also studied. In this study, the delay in initiating ART was the additional time taken for patients between advice of ART by the physician and actual initiation of treatment. Any duration more than one month was considered a delay in starting treatment. Details on alcohol and tobacco consumption and drugs were also collected (Figure).

### Statistical analysis

The sociodemographic and other details are presented as categorical variables (in percentages). Analysis was done using SPSS (version 20.0).

## RESULTS

### Sociodemographic characteristics

In total, 130 patients on ART for at least 6 months were approached, and 100% consented to participate in the study. All the patients were from Puducherry and neighbouring districts of Tamil Nadu, and only one patient was from Andhra Pradesh. The sociodemographic and clinical details of the study population are described in Table 1. Of the total study population, 61% (n=79) were males, and 39% (n=51) were females. Most of the subjects belonged to the age-group of 36-50 years (55%), followed by 21-35 years (31%); 15% were above 50 years of age. Majority (87%) of the respondents were married. Almost half of the participants had received primary education, 47% received up to higher secondary education, and 6.2% (n=8) were uneducated.

Agriculture workers constituted 77% of the subjects while 6% were housewife; 16% were semi-skilled workers, including drivers, construction workers, maids, shopkeepers, and bidi-leaf rollers. Eleven subjects (8.5%) changed their occupation after diagnosis of HIV/AIDS; five shifted to agriculture due to stigma at workplace and ill-health while six subjects were not working due to the illness. One-third of patients (34%) belonged to Class 3 of modified Kuppuswamy's classification, followed by 33% and 23% in Class 2 and 4 respectively; 2% belonged to Class 1, and 8% belonged to Class 5.

**Table 1.** Sociodemographic and clinical details of the respondents (N=130)

Variable	Category	n	%
Age (completed years)	21-35	40	30.8
	36-50	71	54.6
	>50	20	15.4
Gender	Male	79	60.8
	Female	51	39.2
Education class	Uneducated	8	6.2
	Primary school	61	46.9
	Secondary school	49	37.7
	Higher secondary and above	12	9.2
Occupation class	Unskilled	100	76.9
	Semi-skilled	21	16.2
	Skilled	1	0.8
	Housewife	8	6.2
Socioeconomic status*	Class 1 (≤4,678)	3	2.3
	Class 2 (2,339-4,677)	43	33.1
	Class 3 (1,403-2,338)	44	33.8
	Class 4 (700-1,402)	30	23.1
	Class 5 (<700)	10	7.7
Marital status	Married	113	86.9
	Single	17	13.1
Duration since diagnosis (years)	<1	39	30.0
	1-3	71	54.6
	<3	20	15.4
Co-infections**	Yes	89	68.5
	No	41	31.5
Co-morbidities†	Yes	26	20.0
	No	104	80.0

*Socioeconomic status based on Kuppuswamy's socioeconomic status scale—revision for 2011 (17);

**Co-infections, like bacterial, fungal, viral infections, etc.;

†Co-morbidities, like hypertension, diabetes mellitus, peptic ulcer disease, and others

### Clinical details

Half of the participants were diagnosed to have HIV/AIDS between 1 and 3 year(s), one-third within one year, and 15% for more than three years. Two-thirds of the subjects had one or more co-infections—62% had bacterial infections (n=33) and 30% had fungal infections (n=16); two subjects each had viral and other parasitic infections. Of the study subjects, 20% were suffering from one or more co-morbidities (hypertension, diabetes mellitus, peptic ulcer disease, and others). Majority (90.8%) of the respondents were aware of the side-effects associated with ART. Half of the subjects (46.9%) had no side-effects at the time of interview. Gastrointestinal side-effects (38%) included abdominal pain, loss of appetite, diarrhoea, nausea, and vomiting. Nervous system manifestations (7.2%) included numbness, paresthaesia, insomnia, and altered sleep patterns, headache, and vertigo. Dermatological manifestations (2.3%) comprised mostly rashes. Less than 5% of the respondents had adverse reactions involving more than one system.

**Figure:**
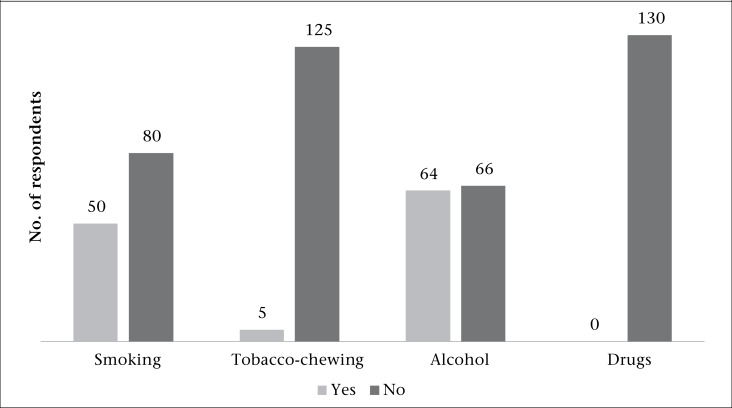
Current alcohol and tobacco consumption among HIV patients at ART centre in Puducherry (N=130)

### Treatment-seeking behaviour

Treatment-seeking behaviour of the subjects (Table 2) was explored as reasons for delay in initiation of ART, reasons for preferring the centre, accompanying persons, travel distance, mode of travel, and expenditure per visit. Family support to the subjects at times of follow-up visit and collection of drugs was also studied. After advice to start ART, there was a delay in starting treatment among one-fifth of the subjects. Reasons for this delay was depression (33%), fear of stigma (33%), fear of disclosure to family (25%), and fear of side-effects (8%). The study elicited various reasons for patients preferring this ART centre—around 55% stated comfort, 41% added confidentiality and privacy, and, for 4%, this was the nearest health facility. All of them collected drugs once a month; 96% found the monthly visits convenient.

One-third of the patients travelled a distance of 31-70 km (38%), and another third travelled less than 30 km. Nearly 11% travelled 70-110 km, and 21% travelled more than 110 km to the ART centre. Although many travelled a long distance to collect drugs, they preferred it because of the lesser chances of public disclosure. However, 41% disclosed difficulty in travelling to the ART clinic; reasons included loss of a day's wage, travelling expenditure, distance, unattended children at home, body aches and fatigue, and fear of stigma. The majority of the subjects travelled to the ART centre by bus (92%) while 8.5% travelled by other means, such as share-auto, bike, etc. Around two-thirds of the patients spent between Rs. 100 and 300 during each visit (including food, travel, and miscellaneous expenditure); one-third spent less than Rs.100, and around 5% spent more than Rs. 300.

### Family support and HIV

Regarding HIV status of spouse, 58% were positive, 38% were negative, and 4% were not tested; spouses of around 9% patients had died. All of the respondents had informed at least one of the members of their families about their disease status. Only 33.8% of the respondents had divulged their disease status to someone other than their immediate family members. Half of the respondents stated that, on disclosure, the family members were initially angry and then turned supportive. In 45% respondents, family members were supportive from the beginning. Less than 1% stated that the family member left home on hearing about the patient's disease. Various ways in which family support was reflected were: reminders to take medication (64%) and motivation to complete treatment (6%). In around one-third of the subjects, families were supportive in many ways—providing motivation, reminding them to take medication, and accompanying them to the ART centre.

**Table 2.** Treatment-seeking behaviour among the respondents (N=130)

Variable	Category	n	%
Duration since treatment (years)	<1	39	30.0
	1-3	71	54.6
	>3	20	15.4
Reason for delay in treatment after advice	Depression	8	33.3
	Stigma	8	33.3
	Fear of side-effects	2	8.3
	Fear of disclosure	6	25.0
Distance of ART centre (km)	≤30	40	30.8
	31-70	49	37.7
	71-110	14	10.8
	>110	27	20.8
Reason for preferring this ART centre	Comfortable	72	55.4
	Nearest health facility	5	3.8
	Confidential (far from home)	19	14.6
	Comfortable and confidential	34	26.2
Accompanying person to the ART centre	Yes	62	47.7
	No	68	52.3
Person collecting ART drugs	Spouse/mother	47	36.2
	Self	83	63.8
Amount spent on travel for single visit (in Rs.)	10-100	44	33.8
	100-299	78	60.0
	>300	8	6.2

Half of the subjects were accompanied by a relative or friend who was HIV-positive. In majority of the cases, the accompanying person was the spouse (89%), followed by a brother (5%), mother (4%), or son (3%). One-third of the respondents also stated that, sometimes, somebody would collect the drugs for them. Most often (72%) it was by the spouse who was also HIV-positive, and this cut down the total travelling expenditure for the month. In a quarter of the subjects, family members, like mother, wife, son, or brother, collected the drugs to ensure that the patient did not lose the wage for the day. In 2% of the respondents, the husband visited the centre instead of the wife who stayed behind to take care of the children.

### Alcohol and tobacco consumption and HIV

Among the subjects, the current use of alcohol was 50%, tobacco-smoking was 39%, and tobacco-chewing was 4%. Majority of the subjects (90%) who cited some form of alcohol or tobacco consumption stated a decrease in these habits since the diagnosis of HIV. This was due to medical advice (56%), fear of death (40%), and independent choice (4%). An increase in their consumption behaviour was seen in 5% respondents mainly due to depression while 5% quit their habits. There was no substance-abuse amongst any of the respondents.

### Perceptions regarding HIV and ART

Stigma, death, and pain were the major concerns about disease progression in 67% respondents. Loss of family support (27%) and inability to support their families (6%) were the other concerns expressed. After initiation of ART, almost all subjects (99%) found a positive improvement in health. Majority of subjects (96%) believed that ART can improve life, and 92% also realized that ART cannot cure their disease. All of them stated high levels of trust and rapport with their doctors and had their apprehensions and doubts clarified. Eight subjects felt a need for improvement in the ART delivery at the centre, like dispensing drugs bimonthly and change in timing of ART clinic.

## DISCUSSION

This study describes the socioepidemiologic profile, treatment-seeking behaviour and perception among HIV patients receiving ART. In this study, the overall male patients outnumbered the female patients—findings similar to sentinel surveillance data and other studies (5-8). Majority of patients were within the age-group of 50 years (86%)—similar to other studies (5-10). The number of subjects who changed or quit jobs due to stigma or illness is less in comparison with other studies.

HIV is increasingly considered a chronic disease. A person living with HIV has to cope with a range of symptoms relating to the infection itself, co-morbidities, or iatrogenic effects from HIV-related medications (11). In this study, it was observed that two-thirds of the subjects had one or more co-infection(s), and one-fifth were suffering from co-morbidities, like diabetes and hypertension. A study done in Surat reported that 66.4% of subjects had opportunistic infections at the time of their first presentation to the clinic (6). The reasons for high prevalence of co-infections in this population are not clear and need to be studied.

In this study, delay in initiation of treatment after advice of ART was seen in one-fifth of the subjects, mainly due to depression, fear of stigma, disclosure to family, and side-effects. A study done in Ethiopia reported low awareness, non-disclosure, perceived side-effects of ART, and HIV stigma as the major barriers for late presentation to HIV/AIDS care (12). HIV patients should be educated that the timely initiation and continuous intake of antiretroviral therapy will not only prolong their survival but will also decrease the viral load and transmission of the disease (10). Understanding the treatment-seeking behaviour of the HIV-positive people on ART, in terms of the reasons for delay in initiation of treatment and the difficulties in accessing the treatment, will help in improving ART facilities and ensuring better linking of PLHA to healthcare.

In this study, one-third of the subjects travelled long distances (more than 100 km) to collect drugs because of the lesser chances of public disclosure. However, they also expressed difficulty in travelling to the ART clinic. Although ART centres are available closer to their residences, patients from far-off places preferred coming to JIPMER to avoid stigma from their community. This was also observed in another study in South India, mainly due to lack of ART centres nearby or avoidance of nearby ART centres due to internalized stigma (7). A NACO study on clients’ perspectives on ART services revealed that distance, travel time, and costs were the main reasons for patients not attending ART services regularly (13). Although majority of PLHA in this study belonged to low socioeconomic status, they spent approximately Rs. 2,400 to 7,200 per year for collecting ART from this link ART centre for confidentiality and comfort. This is despite the fact that facilities were available in proximity to their residence. One possible solution could be the integration of ART services with general medical facilities in such a way that the HIV status of patients is not disclosed to other patients and confidentiality is maintained while providing services. One other measure could be to strengthen post-test counselling by providing information about ART treatment facilities. Measures aimed at increasing the confidence of PLWHA in ART centres, with regard to provision of quality care and resultant improved quality of life can be undertaken through counselling.

In this study, two-thirds of the spouses were HIV-positive, similar to rates found in other studies done in India (7,8). However, less than 5% of them were not tested for HIV status; this is much lower when compared with other centres (8). Many of the HIV patients struggle with numerous social problems, such as stigma, poverty, depression, substance-abuse, and cultural beliefs which can affect their quality of life (QOL) not only from the physical health aspect but also from mental and social health point of view. Social support for patients with HIV/AIDS has shown a strong potential to influence their quality of life. In this study, family members of half of the subjects were supportive from the beginning while the remaining turned supportive after initial phase. Studies have reported that emotionally-sustaining support was considered more desirable and was more often used than other forms of support (11).

Studies have found 68% PLHA having at least any one of the habits, like alcohol, smoking and intravenous drug usage (9). In comparison, alcohol and tobacco consumption in this study was low. However, since the ill-effects of alcohol and tobacco-use are well-known, counselling needs to be undertaken to reduce this behaviour among the study subjects. However, a study in Madurai found that the use of alcohol and smoking was 40% at initiation of ART while it reduced to 7.6% during the study period (7).

The development of combined ART has shifted the perception of HIV/AIDS from a fatal to a chronic and potentially manageable disease. ART is capable of improving survival, reducing the occurrence of HIV-related opportunistic infections, and improving the patients’ QOL (14). In this study, almost all subjects found a positive improvement in health after initiation of ART, and majority believed that ART can improve life. This was also reported in another study where only 0.2% felt no improvement at all (15). Another study reported that the majority of their subjects thought HIV can be controlled by treatment (16). Concerns of clients having children were about the future of their children (7). Counselling services may be arranged to allay their fears and anxiety about the course of the disease. Counselling services can also provide links to other social support mechanisms and community networks for HIV-positive people where they can discuss issues, like future of their children.

PLHA face administrative and procedural problems in hospitals while taking ART, which affect their level of satisfaction with service providers. User's perception about quality of services provided and satisfaction level are guided by factors, like information, access, and guidance; interaction with service providers; physical facilities; confidentiality; discrimination and grievance redressal. In a study done in Chandigarh to assess beneficiary's perception on the quality of services provided at ART centres in India, 88% rated their satisfaction level as satisfactory or above (16). In this study, the subjects reported high levels of trust and rapport with the staff.

### Limitations

Although this study on patients seeking care for HIV in a tertiary-care centre had a very good response rate, understanding of treatment-seeking behaviour from this study is limited to the patients who are currently taking treatment and was not compared with other PLHA in the community, who were not receiving care. Measures of disease progression and immune system response (e.g. CD4 counts, viral load) in these patients are not reported in this study and, hence, their relationship with any other factor was not examined.

### Conclusions

This study among ART recipients reveals several positive aspects, like good family support, better awareness about their disease status, treatment effects, and high levels of support and trust with the doctors and staff. However, a few issues highlighted in this study, like alcohol and tobacco-use, travelling long distance for drug collection, fear of stigma and death, and concerns regarding the future, need to be addressed.
